# Clinical characteristics and predictors of mortality in patients with normal wall thickness cardiac amyloidosis

**DOI:** 10.1186/1532-429X-18-S1-P131

**Published:** 2016-01-27

**Authors:** Sabha Bhatti, Evan Watts, Srikanth Vallurupalli, Tarun Pandey, Abdul Hakeem

**Affiliations:** Cardiology, UAMS, Little Rock, AR USA

## Background

Clinical features and prognosis of Cardiac amyloidosis (CA) with normal wall thickness (NWT) have not been well elucidated.

## Methods

305 patients with multiple myeloma undergoing cardiac MRI for suspected amyloidosis were consecutively enrolled. 251 patients underwent gadolinium study. Increased wall thickness was defined as left ventricular wall thickness >12 mm on CMR.

## Results

74 patients (29%) had gadolinium enhancement pattern consistent with CA on CMR (31% with NWT). With endomyocardial biopsy as the gold standard, amyloid pattern on CMR (LGE+) had sensitivity and negative predictive values of 100%; specificity and positive predictive values of 80% and 81% with an AUC 0.9 for CA. There was no difference between CA patients with NWT and increased wall thickness in age (60 vs. 62 years; p = 0.56);) EF (60+11 vs 59+13;p = 0.7) IgA (72 vs. 56;p = 0.07); hypertension (52% vs. 33%; p = 0.8),diabetes (17% vs. 22%; p = 0.9);chronic kidney disease (43% vs 28%; p = 0.2) and low voltage EKG (23% vs. 26%; p =0.7). There was a difference between the two groups in the following variables:female gender (60% vs. 23%; p-0.007), WT (0.97+0.1 vs. 1.5+0.2;P < 0.0001) EDV (109+55 vs 86+45;p = 0.05); BNP (249 vs 629;p = 0.01); lambda light chains (6.8 vs 2.6; p = 0.04). There was no difference in the median survival between the two groups (50%vs. 52%; log rank p value=0.9); median survival times (1215 vs. 1450 days; p =0.6).

## Conclusions

NWT CA has unique clinical and biochemical profile compared to patients with increased wall thickness CA. Since prognosis is no different than patients with increased wall thickness CA, LGE CMR and biochemical markers can help in early detection and risk stratification of this high risk subgroup.

